# Prevalence of eating disorders in adolescent girls in Siberia

**DOI:** 10.1192/j.eurpsy.2022.548

**Published:** 2022-09-01

**Authors:** N. Semenova, H. Slobodskaya, E. Rezun

**Affiliations:** 1 Scientific Research Institute of Medical Problems of the North, Department Of Child’s Physical And Mental Health, Krasnoyarsk, Russian Federation; 2 Federal State Budgetary Scientific Institution «Scientific Research Institute of Neurosciences and Medicine», Department Of Child Development And Individual Differences, Novosibirsk, Russian Federation

**Keywords:** Epidemiology, Adolescents, Eating Disorders, girls

## Abstract

**Introduction:**

Eating disorders (ED) are an urgent public health problem, however, many adolescents with clinical symptoms fail to meet stringent diagnostic criteria.

**Objectives:**

To estimate the prevalence of eating disorders (ED) and subthreshold eating disorders (SED) in adolescent girls.

**Methods:**

A cross-sectional study of girls attending secondary schools (n = 917) was carried out. The sample comprised of 18.3% early adolescents (aged 12-13), 51% middle adolescents (aged 14-15), and 30.6% late adolescents (aged 16-17). We used the Body Image and Eating Distress scale (Koskelainen et al., 2001) coded on a 1-3 scale. The answers were scored on a scale of 1-3. Adolescents scoring 12 or above on four items measuring body dissatisfaction were considered as dissatisfied with their bodies and were further divided into two subgroups: girls scoring 10 or above on three items measuring eating distress were considered as having ED, whereas girls scoring less than 10 were considered as having SED.

**Results:**

The prevalence of ED was 2.1% (CI 1.4-3.3), the prevalence of SED was 9.6% (CI 7.8-11.7). In early adolescence, the prevalence of SED was 1.6% (CI 0.9-2.7). In middle adolescence, the prevalence of SED was 5.1% (CI 3.9-6.7), the prevalence of ED was 0.9% (CI 0.5-1.8). In late adolescence, the prevalence of SED was 2.8% (CI 1.9-4.1), the prevalence of ED was 1.2% (CI 0.7-2.1).

**Conclusions:**

In adolescent girls, the SED are 4.6 times commoner than overt above-threshold ED. During adolescence, the prevalence of SED decreases, while the prevalence of ED increases with age.

**Disclosure:**

The reported study was funded by grant RNF according to the research project № 21-15-00033

